# Small Bowel Diverticulosis in an Elderly Patient Presenting With Obstruction and Intestinal Dysmotility: A Case Report

**DOI:** 10.7759/cureus.71422

**Published:** 2024-10-14

**Authors:** Annie Tonnu, Jonathan Henning, Salvatore Docimo, Christopher G DuCoin, Joseph A Sujka

**Affiliations:** 1 Department of Surgery, Wayne State University School of Medicine, Detroit, USA; 2 Department of Surgery, University of South Florida Morsani College of Medicine, Tampa, USA

**Keywords:** internal hernia, jejunal diverticulosis, laparoscopy, laparotomy, pseudo-obstruction, small bowel diverticulosis, small bowel resection

## Abstract

Small bowel diverticulosis can occur anywhere in the small bowel but is most common in the duodenum. Jejunoileal diverticulosis is much less common and tends to have a more chronic, nonspecific disease course. In the literature, this condition has a higher incidence in men in their sixth and seventh decades of life. Diagnosis of small bowel diverticula can be difficult and may depend on surgical exploration of the bowel content to confirm the final diagnosis.

An 81-year-old elderly female patient with no past surgical history presented with chronic abdominal pain, distension after eating, occasional diarrhea, and weight loss. Her initial presentation was concerned with pseudo-obstruction and possible internal hernia. She had multiple rounds of antibiotics to diagnose small intestinal bacterial overgrowth (SIBO). After a diagnostic laparoscopy due to persistent symptoms, the diagnosis of small bowel diverticulosis was confirmed. The patient ultimately decided to undergo a laparotomy with a small bowel resection to treat her condition. Her postoperative course was complicated by an episode of Giardiasis, which was treated with a course of antibiotics. Upon her follow-up appointment in the clinic, she reported doing well without any complaints.

While small bowel diverticulosis can be asymptomatic, a presentation of persistent symptoms, including generalized abdominal pain, diarrhea, and distension, can occur, especially in the older population of patients. Diagnosing this condition can be challenging and usually can be accomplished through laparoscopy. However, definitive treatment leading to the cessation of symptoms can only be achieved through surgical resection of the involved segment of the bowel.

## Introduction

Diverticula are pouch-like protrusions that can occur along the gastrointestinal tract through weak points in the muscular wall [1,2]. They are mostly found in the colon, and their relevance increases with age. Diverticulosis is the condition defined by the presence of diverticula. Diverticula can be divided into true and false types. A true diverticulum includes all the intestinal layers, such as Meckel's diverticulum, while a false one only consists of mucosal and submucosal layers. Most people with diverticulosis are asymptomatic [3]. If left untreated, symptoms can form, including generalized abdominal pain, diarrhea, and signs of intestinal obstruction. Infection of the diverticula due to stool burden can cause bleeding and inflammation, termed diverticulitis [1].

Small bowel diverticulosis can occur anywhere in the small bowel and is less common than colonic diverticulosis. It is most often found in the duodenum and has a low incidence of complications [3,4]. However, jejunoileal diverticula, despite being rarer, have a higher complication rate, sometimes leading to the necessity of an operative approach as the treatment. Thus, clinical recognition and diagnosis are important to prevent the occurrence of diverticulitis, which has a mortality rate as high as 24% [1].

We report a case of symptomatic jejunal diverticulosis with a challenging course of diagnosis as well as medical and surgical management to reduce the risk of complications.

## Case presentation

An 81-year-old female patient with a medical history of osteoporosis and saddle pulmonary embolism (on Apixaban) presented to our outpatient clinic with generalized abdominal pain and distension associated with eating. These symptoms had been persistent for about seven years. She had received various treatments relating to collagenous sprue, celiac disease, and small intestinal bacterial overgrowth, without relief. Her CT scan in March 2024 showed almost the entirety of the jejunum and ileum were fluid-filled, perhaps light wall thickening and enhancement, with a few loops dilated up to 26 mm, but not pathologically dilated. There was no definitive acute inflammation or hyperemia in the adjacent small bowel mesentery. A transition point was associated with the vascular swirling of the central small bowel mesentery. There was a transition between dilated and non-dilated loops at this location, suggesting an internal hernia.

The patient had not had a surgical evaluation of her abdomen until this visit. At this point, we discussed with her that the next step would be a robotic exploration to further evaluate the internal hernia and close the defect if possible. Between the initial visit in April and the robotic exploration in May, the patient reported weight loss, loose stools, and abdominal pain concerning for a partial bowel obstruction.

The patient had a diagnostic robotic laparoscopy. During the procedure, we noted diverticula in the patient's small bowel. We were initially concerned that it could be Meckel's diverticulum, but it became apparent that there were many more than just a single diverticulum. The largest population of diverticula was in the mid-jejunum. There was obvious peristalsis in the small bowel. The diverticula did not have any appearance of cancerous lesions and was not firm in any way. An internal hernia was not identified. A diagnosis of small bowel diverticulosis was established.

The patient did not consent to exploratory laparotomy and bowel resection and after an intraoperative discussion with the family, the laparoscopy was aborted and the case ended. The patient recovered without any complications after the diagnostic laparoscopy. On day seven after the diagnostic laparoscopy, she presented in the emergency department with worsening oral tolerance and abdominal pain. A repeat CT scan showed diffuse pneumatosis intestinalis and pneumoperitoneum concerning for ischemia with perforation as well as wall edema of a loop of jejunum with probable focal perforation. The patient was immediately admitted to the general surgery floor and kept nil per oral (NPO) with a low suspicion for perforation based on clinical evaluation, including a benign abdominal examination.

The patient complained of new-onset diarrhea. Gastrointestinal specialists were consulted for the diarrhea with recommendations for a diarrhea workup, an esophagogastroduodenoscopy with duodenal biopsies, and a colonoscopy. Small bowel biopsies displayed intraepithelial lymphocytosis without villous atrophy. This was not consistent with celiac disease, and tissue transglutaminase was negative. Pathology did not appreciate a significant collagen band, ruling out collagenous sprues. Jejunal aspirates with heavy growth of Gram-negative bacteria that had speciated to *Klebsiella* and *Kluyvera*, confirming small intestinal bacterial overgrowth (SIBO). The patient was informed that the treatment options include antibiotics and small bowel resection. She was also informed that neither was guaranteed to relieve her symptoms, but the GI specialists recommended trialing antibiotic therapy that was directed to her culture sensitivities and may continue to discuss potential small bowel resection as an outpatient. The workup for diarrhea was negative, including the *Clostridioides difficile* (*C. diff)* toxin and GI pathogen panel. Thyroid-sTSH was unremarkable. Fecal elastase was within normal limits. Calprotectin was the only test that was elevated. The patient was treated conservatively, and discharged home.

During a follow-up appointment in the clinic, the patient reported persistent diarrhea and lost approximately 5 lbs. in two weeks. After a long discussion of possible treatment options, the patient elected to proceed with an exploratory laparotomy with small bowel resection, and possible antibiotics after surgery. She decided against another antibiotic course because this would have been her fourth round of antibiotics, and she did not like to proceed with ongoing antibiotics especially given her recent diarrhea.

**Figure 1:**
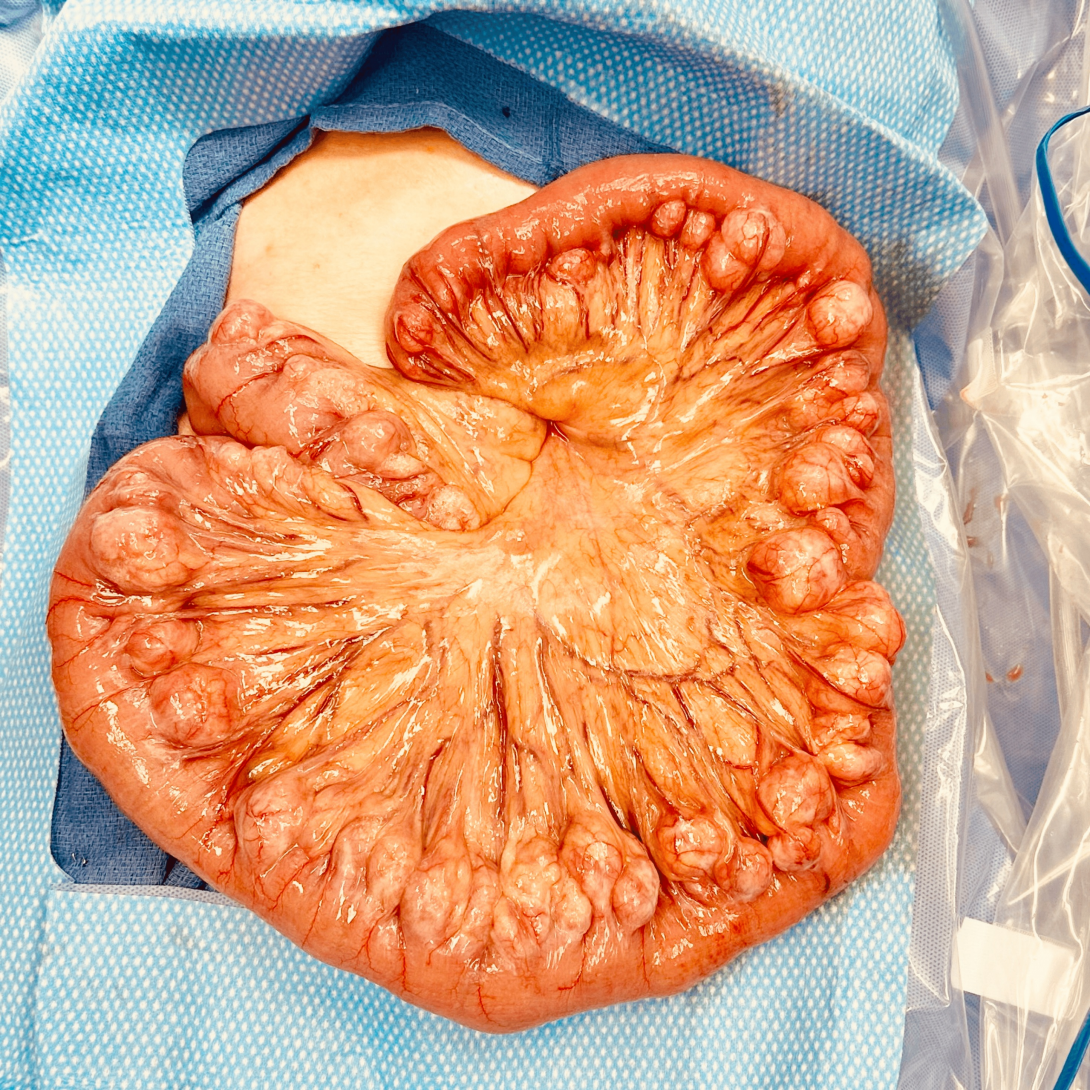
Exploratory laparotomy showing multiple small bowel diverticula within the jejunum of the 81-year-old female

During the procedure, it was apparent that there was a large segment of the small bowel with innumerable diverticula in the mid-jejunum (Figure 1). The area of diverticulosis began approximately 20 centimeters from the ligament of Treitz and continued throughout the jejunum. After confirming that the patient had enough healthy bowel distally (> 100cm), we elected to remove the entire diverticular segments. The patient’s postoperative course was unremarkable, and she was discharged two days after the procedure.

She was discharged home on antibiotics and was told to follow up in the clinic in two weeks. During her follow-up visits, she reported no new complaints and felt well overall.

## Discussion

Diverticulosis of the jejunum and ileum is uncommon, with a reported prevalence of 0.3% to 1.9% on conventional barium studies and 0.3% to 1.3% on autopsies. The cause of jejunoileal diverticulosis remains unclear, but intestinal dyskinesia, peristaltic abnormality, and high intraluminal pressure are thought to play a role in developing this condition [3].

Although small bowel diverticula are usually asymptomatic and are discovered incidentally, the complications might be severe if left untreated. Jejunoileal diverticula are often rarer with the diagnosis being coincidental in 70% of patients [5] and can present with vague symptoms, such as diarrhea, abdominal distension, and sometimes signs of bowel obstruction such as our patient’s chronic symptoms. Generally, there is no standardized test to confirm jejunoileal diverticula. Therefore, a clinical picture can be confused with other inflammatory conditions, such as appendicitis, cholecystitis, and acute colonic diverticulitis, highlighting the challenges in diagnosing this condition [1]. Computed tomography (CT) and endoscopy may help rule out inflammatory processes and other small bowel disorders. However, a conclusive diagnosis can only be made with a diagnostic laparoscopy, and a definitive treatment can be achieved through an exploratory laparotomy and small bowel resection of the involved area [3].

Compared to duodenal diverticula, jejunoileal diverticula are four times more likely to develop a complication and are 18 times more likely to perforate and develop abscesses [2]. Therefore, a prompt diagnosis and treatment plan is necessary to prevent morbidity and mortality. For patients with chronic symptoms, like the patient in our case, conservative treatments can be tried. If unresponsive, a more aggressive surgical approach is justified, especially if there are signs of complications [1].

There are challenges in diagnosing chronic abdominal pain and distension, especially with overlapping conditions like collagenous sprue, celiac disease, and SIBO. Our patient had concomitant culture-proven SIBO, which had been refractory to several rounds of antibiotic treatment. Though the co-existence of SIBO and small bowel diverticula is not necessarily uncommon, it complicated the approach for diagnosis and treatment, i.e. antibiotics versus surgery [6], especially in elderly patients with comorbidities. Our patient’s symptoms were severe and refractory to other treatments, so even though surgery was the last resort, the potential for improved quality of life exceeded the risk.

## Conclusions

There are challenges in diagnosing chronic abdominal pain and distension, especially with overlapping conditions like collagenous sprue, celiac disease, and SIBO. Our patient had concomitant culture-proven SIBO, which had been refractory to several rounds of antibiotic treatment. Though the co-existence of SIBO and small bowel diverticula is not necessarily uncommon, it complicated the approach for diagnosis and treatment, i.e. antibiotics versus surgery, especially in elderly patients with co-morbidities. Our patient’s symptoms were severe and refractory to other treatments, so even though surgery was the last resort, the potential for improved quality of life exceeded this risk.
